# An Online Database of Infant Functional Near InfraRed Spectroscopy Studies: A Community-Augmented Systematic Review

**DOI:** 10.1371/journal.pone.0058906

**Published:** 2013-03-15

**Authors:** Alejandrina Cristia, Emmanuel Dupoux, Yoko Hakuno, Sarah Lloyd-Fox, Manuela Schuetze, José Kivits, Tomas Bergvelt, Marjolijn van Gelder, Luca Filippin, Sylvain Charron, Yasuyo Minagawa-Kawai

**Affiliations:** 1 Neurobiology of Language, Max Planck Institute for Psycholinguistics, Nijmegen, The Netherlands; 2 Laboratoire de Sciences Cognitives et Psycholinguistique, Ecole des Hautes Etudes en Sciences Sociales and Ecole Normale Supérieure, Paris, France; 3 Graduate School of Human Relations, Keio University, Tokyo, Japan; 4 Centre for Brain and Cognitive Development, Birkbeck, University of London, London, United Kingdom; 5 Institut d'Etude de la Cognition, Ecole Normale Supérieure, Paris, France; Lancaster University, United Kingdom

## Abstract

Until recently, imaging the infant brain was very challenging. Functional Near InfraRed Spectroscopy (fNIRS) is a promising, relatively novel technique, whose use is rapidly expanding. As an emergent field, it is particularly important to share methodological knowledge to ensure replicable and robust results. In this paper, we present a community-augmented database which will facilitate precisely this exchange. We tabulated articles and theses reporting empirical fNIRS research carried out on infants below three years of age along several methodological variables. The resulting spreadsheet has been uploaded in a format allowing individuals to continue adding new results, and download the most recent version of the table. Thus, this database is ideal to carry out systematic reviews. We illustrate its academic utility by focusing on the factors affecting three key variables: infant attrition, the reliability of oxygenated and deoxygenated responses, and signal-to-noise ratios. We then discuss strengths and weaknesses of the DBIfNIRS, and conclude by suggesting a set of simple guidelines aimed to facilitate methodological convergence through the standardization of reports.

## Introduction

Until recently, it was extremely challenging to neuroimage the emergence of functional brain regions and networks in the infant brain. This is quickly changing with the advent of new and improved technologies. In particular, functional Near InfraRed Spectroscopy (fNIRS) research is rapidly growing, as a relatively inexpensive and child-friendly technique, which can reveal functional activation and functional connectivity between different cortices.

To briefly summarize some key features of the technique, fNIRS provides an index of local changes in blood volume, estimated through the absorption of near infrared light traveling between a given source and a detector. As a hemodynamic method, it provides unambiguous evidence that changes in hemoglobin concentration occur in a circumscribed volume located between the source and detector. However, since spatial localization can only be done by reference to superficial landmarks, one cannot ensure which precise cortical areas have been measured. Additionally, areas targeted are limited to surface structures, as it is unlikely that deep regions, such as the amygdala, may be reliably measured. As in functional Magnetic Resonance Imaging, one can analyze correlations between independent channels within low-frequency bands as an index of functional connectivity. Since fNIRS does not depend on electrical signals, it is more resistant to artifacts that would be caused by blinking or moving one's limbs. Its resistance to motion artifacts in general is one of the commonly cited advantages, although we revisit this question below. As a result of an increasing interest in fNIRS as a technique to image the infant brain, numerous technical introductions have been published recently. Readers who would like to learn more can turn to any of a number of introductions to infant fNIRS [1]–[5].

The number of research laboratories that have recently acquired, or are considering acquiring, a system to do fNIRS is remarkable. However, even for experienced fNIRS researchers, it is still unclear how to select some important methodological details. Many such methodological questions have been the center of discussion in the infant fNIRS community, of which we give three examples: What wavelengths should be used? How many sources and detectors can be incorporated, and at what distance should they be placed? Are there systems that are more effective with specific age groups? Clearly, some of these questions would be ideally answered through careful pairwise testing, experimentation, and forward modeling of light transport in brain tissues. However, while the community gathers enough principled evidence, a first approach is to estimate the most appropriate empirical answers on the basis of existing research. In other words, while we may not yet understand why certain parameter combinations ‘work better’ than others, we might be able to share this knowledge with others, to help them avoid the same errors we have made.

Our goal was to pool such indirect information on the performance of specific methodological choices into a database, from which both newcomers and experienced users can draw empirical generalizations that may inform their know-how. To this end, we tabulated studies that had previously been published (see below for details on the inclusion criteria); but we wanted to go beyond a meta-analysis. In such a quickly growing field as infant fNIRS, we deemed it likely that whatever generalization we may draw now might soon become either limited (because it did not take into account additional factors) or incorrect (as new studies may show otherwise). Therefore, we made use of tools that are currently freely available to create an online resource that can grow through independent contributions in a minimally supervised manner.

In this report, we first introduce how we compiled studies into the database (henceforth DBIfNIRS). Thereafter, we explain how consumers of fNIRS literature can download the spreadsheet containing all coded studies, and describe more specifically how producers of infant fNIRS literature can make use of an online form in order to add recently published studies into the database. Then, we provide three examples of how DBIfNIRS can be used to inspire methodological research by experts, and to facilitate methodological decisions by users. The first case study suggests that attrition (i.e., infant exclusion due to non-completion of the paradigm or poor data quality) remains a considerable roadblock to infant fNIRS research, although some age groups and technical configurations seem more promising than others. The second case study investigates the highly debated question of whether oxygenated, deoxygenated, and total hemoglobin measures are equally reliable. The third assesses whether study design features pertaining to study duration lead to better results. We then summarize the strengths and weaknesses of this database, which could be ameliorated through more consistent reports in the literature. To this end, we lay out a guideline for infant fNIRS reporting in the final section.

## Methods

### 1. Compiling DBIfNIRS

Empirical research in English reporting fNIRS data from infants (0–36 months of age) were identified by: (1) doing searches for [infant {fNIRS | optical topography | NIRS}] on scholar.google.com; (2) finding papers that cited foundational studies or reviews (e.g., [1], [6]); (3) tracing back papers cited in the bibliographies of all the work found through (1) and (2). When likely candidates were found, they were inputted in a list. Then one of the authors read the abstract and, if necessary, the methods to confirm that the study used fNIRS and was ran on an infant group. Only citable papers (journal articles and theses) were considered. There were no other criteria for exclusion.

We then compiled a set of instructions detailing inclusion/exclusion criteria and what key information to enter. Table 1 shows the variables that were tabulated, and the explanations for each. At a subsequent stage, each study was assigned to one of the authors, who read the methods in great detail and the rest of the paper for reference. This coder also entered key information following the main instructions. When instructions were not clear or in case of doubt, they consulted the first author, and instructions were re-written. After the spreadsheet was complete, the first author looked through all entries in order to detect aberrant entries or inconsistent criteria. When odd entries were found, we re-read the original paper and corrected the entry.

**Table 1:** Variables coded.

**Data Grouping**	Each line/entry represents one group of data, which are grouped on the basis of participants. If there are more than two groups of infants reported, write here a code indicating how these groupings are made (i.e., group1_3mo = this line refers to the first group, which are 3-month-olds). If there is only one group of infants reported, write ‘single’.
**Infant type**	What kinds of infants were tested? Make your choice between: standard (i.e., healthy and fullterm), preterm (but healthy), mixed (if the grouping was not strict, so there are preterms and fullterms, healthy and not healthy), pathologic (if the infants had *any* neurological condition AND/OR were provided with drugs)
**Infant type details**	Provide more specific details; e.g. healthy_full-term_neonates, infants_with_mild_hypoxic-ischemic_encephalopathy_(HIE)
**Infant state**	Choice between: awake, asleep, mixed
**Apgar**	What criterion in terms of the 5 minute apgar score did the authors use? *Note: few studies reported this, so this variable has been removed from the current DBIfNIRS*.
**Drugs**	Were any drugs given to the participants? *Note: few studies reported this, so this variable has been removed from the current DBIfNIRS. Sedated infants have been incorporated to the ‘pathologic’ type above*.
**Number of infants**	Number of infants that have been included in the analyses
**Boys**	Number of included male infants
**Girls**	Number of included female infants
***Age Group***	*This variable is typically filled out with a formula, using the age in Avg_CA; if no average age was given, then it was filled out by hand from the authors*' *description*.
**Average Chronological Age**	Average age in days from birth. This is typically reported as ‘age’; when infants are premature, if ‘corrected age’ or ‘maturational age’ are reported, their chronological age = corrected age + (280–gestational age [time in the womb]).
**Minimum Chronological Age**	The lower bound in the range of chronological age in days
**Maximum Chronological Age**	The upper bound in the range of chronological age in days
**Average gestational age at birth**	Average gestational age at birth in weeks (if the paper says ‘full term’ and doesn't report exact GAB, then write NA)
**minGAB**	The lower bound in the range of gestational age at birth in weeks
**maxGAB**	The upper bound in the range of gestational age at birth in weeks
**nbSExcluded**	Number of infants that have been excluded in the analyses
**Criteria for exclusions**	Why were those infants excluded?
**System**	Which brand of fNIRS system was used?
**SystemDetails**	Details of fNIRS system, such as the model name/number and generation
**wavelengthLowernm**	Lower wavelength in nm
**wavelengthHighernm**	Higher wavelength in nm
**wavelengthOthernm**	Any other information on wavelength (i.e. other wavelengths if more than 2 were used)
**Power_mW**	Average power per physical source in mW
**padOverLobe**	Which broadly defined area did the physical pad cover? Choice between: frontal, temporal, occipital, parietal, multiple (if a single physical pad probably spanned multiple lobes, or multiple lobes spanning single areas were covered)
**CorticalPrecise**	What specific brain areas did the authors claim were targeted by the pad? (i.e. inferior frontal, superior frontal, inferior temporal, superior temporal, posterior temporal, and temporo-parietal)
**Sources**	Total number of light sources
**Detectors**	Total number of detectors
**Channels**	Total number of channels investigated
**ChanSep_mm**	What was the separation between adjacent sources and detectors?
**Stimuli.General**	What general type of stimulation was used? Choice between: audio, audiovisual, motion, odor, pain, touch, visual, other
**Processing.Focus**	What was the type of processing under study according to the authors? Choice between: action, audiovisual, auditory, emotion, events, faces, language, motor, music, numeric, object permanence, odor, pain, social, visual, voice, other
**Stimuli.specific**	The specific description of their stimulation
**DurationBlocks_s**	If the research uses block designs, how long was a single stimulation or a stimulation train in seconds?
**MinNumberBlocks**	What was the minimum number of blocks that infants had to complete in order to be included in the analyses?
**MaxNumberBlocks**	What was the maximum number of blocks that infants had completed?
**Baseline**	What happened while no stimulation was being presented?
**minDurationBaseline_s**	What was the minimum duration of the baseline in seconds?
**maxDurationBaseline_s**	What was the maximum duration of the baseline in seconds?
**Methods**	Any other methodological detail that was not covered in the previous questions (e.g. low- and high-pass filtered)
**Oxy.1increase**	How did oxy-Hb concentration change as a function of stimulation according to the authors? Choice between: 1, 0, −1
	1: increase
	0: no clear trend in either direction
	−1: decrease
**Oxy_details**	Any other details regarding the behavior of oxy-Hb as a function of stimulation; also enter here a note if the description above is taken from a selection of infants, or a selection of channels; time to peak was XX, average signal to noise ratio, an unusual statistic etc.
**Deoxy.1decrease**	How did deoxy-Hb concentration change as a function of stimulation according to the authors? Choice between: 1, 0, −1
	1: decrease
	0: no clear trend in either direction
	−1: increase
**Deoxy_details**	Any other details regarding the behavior of deoxy-Hb as a function of stimulation.
**Tot.1increase**	How did total-Hb concentration change as a function of stimulation according to the authors? Choice between: 1, 0, −1
	1: increase
	0: no clear trend in either direction
	−1: decrease
**Total_details**	Any other details regarding the behavior of total-Hb as a function of stimulation
**otherHRFdetails**	Any other details regarding the hemodynamic response registered
**otherDetails**	Any other considerations on the study, its design, its participants, its results
**Figures**	Keep copies of figures representing the hemodynamic response function. *Note: the type of figure reported is too variable across studies to provide bases for any reasonable generalizations, and this item was removed from the database*.
**Abstract**	Copy the whole abstract

At the following stage, we revisited the spreadsheet and instructions, simplifying them for use by the community at large. This version was uploaded to docs.google.com and a form was created. The website nucleates these materials, and it is associated with a blog where users can post comments to alert of inaccuracies and managers can announce changes to the database (such as corrections). As a final check, the website address was provided to 3 experts who have published in the infant fNIRS field, and they were asked to enter their latest paper, with no further instructions provided. This helped hone the interface and instructions. It should be noted at no stage did we assess risk of bias in individual studies. Since the database was intended to be a community resource, assessing bias for previous studies seemed unreasonable. The full PRISMA checklist and diagram are provided as Table S1 and Figure S1.

### 2. Using DBIfNIRS: Consumers, contributors & supervisors

Accessing and contributing to DBIfNIRS does **not** require one to have a google-registered address, since all of the following sites are open to anyone on the public web (provided access to google documents is allowed). To **download** DBIfNIRS, one may simply visit https://sites.google.com/site/dbifnirs/and click on the appropriate links. The links are automatically updated to the most recent version posted by the supervisor. Users should first of all read the comments in the associated blog (http://dbifnirs.blogspot.nl/), which will indicate any errors that have already been spotted by fellow users, but not yet corrected by the supervisor. In a project of this size, it is possible that there be a small proportion of errors. If possible, users should post messages to the blog to inform the community of any errors they observe, which can be done anonymously without a google-registered account; and the supervisors will notify the community through the same medium once the errors have been corrected. They can also email one of the supervisors, whose email will be clearly stated on the DBIfNIRS website.

One can also **contribute** to DBIfNIRS by entering new studies using the form that is accessible from https://sites.google.com/site/dbifnirs/. In order to contribute, we **strongly recommend** to first read through previous entries, as well as the online instructions and the comment forum, so as to ensure that their entry is consistent in criteria with those of previous contributors. To avoid frustration through dropped half-completed entries, producers should also make sure they have all the information they need before they start completing the form. Previous users have estimated it takes between 30 minutes and 2.5 hours to enter one paper.

The database can grow and prosper thanks to the attention and contribution of the community. However, to remain up to date and trustworthy, DBIfNIRS requires someone who corrects errors that have been spotted by the users, and, when needed, stimulates and ensures data entry. Therefore, the database will always have two supervisors, expert volunteers who will serve first as trainee, then as main database supervisor, each time for a period of one year.

## Results

We first draw a general overview of the studies entered at the writing of this article (section 4.1). In addition to providing a tally of the conceptual issues tackled using infant fNIRS field, DBIfNIRS also allows an initial exploration of a number of methodological choices. We chose three case studies to illustrate this, discussed in following subsections (sections 4.2–4.4). Finally, inspection of a sample of studies revealed that, at present, tabulating pre-processing and analyses methods is not feasible for the field, due to the large variability in the actual methods used, and in the manner of reporting.

### 1. General overview

On July 20, 2012, DBIfNIRS included 76 articles or theses, reporting results from 3557 infants distributed into 109 entries (which are defined on the basis of infant groups; see previous section for details). An additional 8 articles had been shortlisted but were discarded because they contained no fNIRS data (e.g., reviews). For simplicity, we provide all numbers as percentages of entries. By far, the most common focus of interest was language (25%), followed by processing of auditory (13%), visual (12%) and face stimuli (11%). Other types of stimulation which made up at least 3% of the sample were motor, music, social, and pain. About 3% of the studies did not contain any external stimulation, as they focused on correlations across channels tapping different brain areas or the relationship between neural events recorded through EEG and fNIRS.

The overwhelming majority of studies are carried out with fullterm, healthy infants (87%). Six percent of entries correspond to healthy preterm infants and another 6% to infants with a diagnosis of neurological problems or difficult birth. Few studies have sample sizes greater than 40 in any infant group, with the median of infants included being 15.

**Figure 1:**
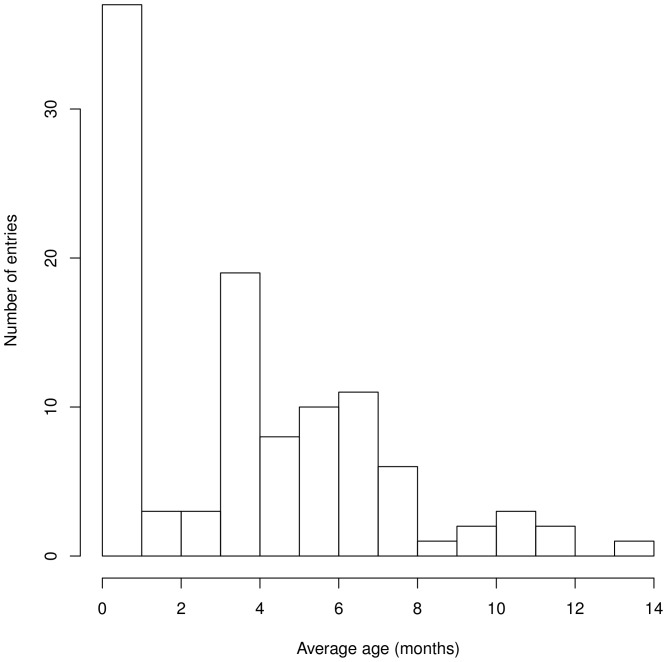
Age groups studied. The upper boundary of the histogram has been fixed to exclude the outlier at 27 months.

Aside from an outlier (an entry of 27-month-olds), most studies are carried out on young infants. As shown in Figure 1, the distribution of studies per age group is bimodal. Newborn studies make up 32% of the entries. There is a second cluster of age groups tested, starting from 4-month-olds (the second most commonly studied age group, 17% entries) to about 8 months of age. The right tail of the distribution stops abruptly at 14 months. This may reflect a limitation in the practical aspect of testing toddlers with methods that require a certain degree of immobility and infant compliance.

By far, the most commonly used brand of system is Hitachi (58%). The second most common is Hamamatsu (20%). There were only 3 other system brands used (ISS, Shimadzu, UCL), each represented in less than 5% of the entries. About 5% of the entries used an in-house system, and another 5% did not report what kind of system was used.

Most entries used 2 wavelengths, of which the lower was either around 780 nm, or around 690 nm. The latter only occurs in studies after 2004, reflecting the adoption of a wavelength at around 690 nm in one of the Hitachi models. The higher-end wavelength is most commonly around 830–850 nm, which is not independent from the disproportionate incidence of Hitachi systems in current data. We return to the question of wavelength in our second case study reported below.

Most studies (28%) focus on temporal areas. An exclusive focus on frontal (16%) and occipital (10%) is not rare, whereas no study taps solely parietal regions. This may relate to the fact that most studies focus on language and auditory regions, and these researchers may tend to target specific structures that in adults are commonly associated with processing such stimuli. In addition, the ease with which one can design headgear that targets a specific region of interest varies considerably given the limitations inherent to fNIRS. The scalp over temporal regions is flat, making it easy to design pads that will fit most infants, and the ears are a prominent landmark, facilitating more accurate optode placement. Anterior frontal areas are free of hair and the nasion serves as a reference point. Finally, occipital areas are easily identified with surface landmarks such as the inion. In contrast, parietal regions are at a disadvantage on all of these counts. There is hair, no clear surface landmarks, and the scalp curves, making it difficult to design a headgear that will sit comfortably on most infants' heads. It is extremely common to find studies with pads spanning multiple regions or with multiple pads, allowing more or less independent coverage of two or more regions. A non-negligible cluster of studies (10%) has been carried out with a very dense cap, sampling from all sides of the head.

**Figure 2:**
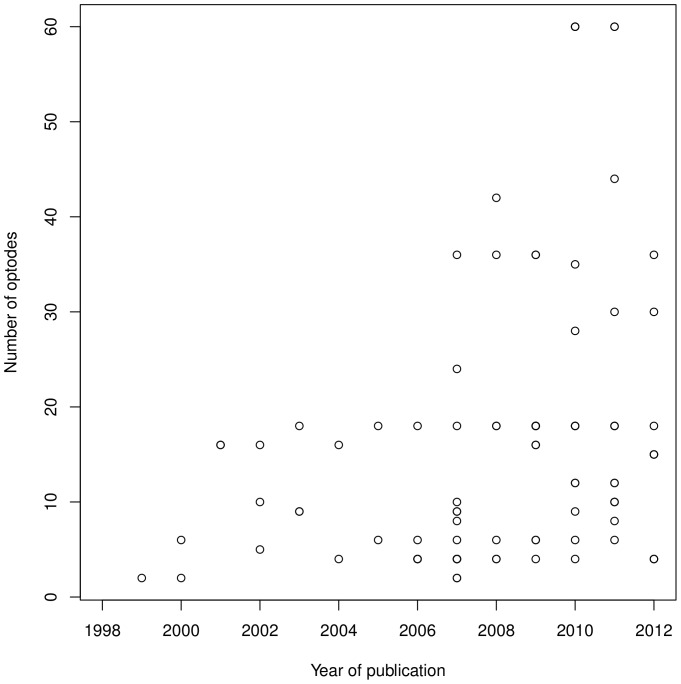
Number of optodes as a function of year. Number of optodes is totaled over sources and detectors.

There has been a clear trend to incorporate a higher number of sources and detectors over the years, as illustrated in Figure 2. Source and detectors were most commonly separated by 40 mm before the year 2000, but subsequently this separation was typically in the range between 20 and 30 mm, only in rare studies exceeding 35 mm. A recent trend is to use multiple separations within the same study, which is found in less than 4% of the sample. This can be done easily in systems that provide measurements for all source-detector pairs (e.g., UCL, but not Hitachi), since then pads can be designed such that a given source will reach 2 or more detectors at different distances. Whether there is an ideal separation to use continues to be debated [5], [7], and using multiple separations allows researchers to sample from different volumes with little extra cost (as exemplified in [8], [9]). At present, DBIfNIRS cannot easily capture such designs, but future versions may have to contemplate this possibility if multi-separation caps become more prevalent.

### 2. Case study 1: Attrition rate

One of the most prevalent problems in infant research is data loss due to infant non-compliance. This problem is already considerable in brief behavioral studies, which do not require much of the infant, and it could be aggravated in procedures that are longer and require infant ‘manipulation’ (to place a cap on them), such as EEG and fNIRS. In addition, even if infants are content until the end of their study, they might not hold sufficiently still, and their data may have to be excluded because of the prevalence of artifacts. Since this is a topic of crucial interest to infant researchers, we sought to uncover the variables that best predict participant attrition, such that these factors may be taken into account in future research.

**Figure 3:**
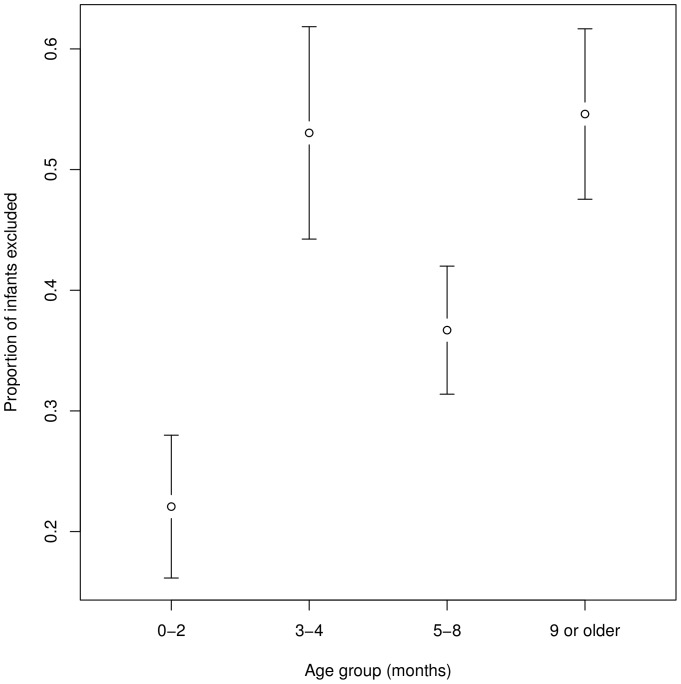
Proportion of infants excluded by age group. Error bars indicate 95% confidence intervals.

It is remarkable as well as unfortunate that 30% of the sample did not report attrition data. There was no relationship between attrition rate and infant state (awake or asleep). In contrast, there were clear differences depending on the age group studied. As Figure 3 shows, the lowest attrition rates are found in newborns and in 5- to 8-month-olds. While it is unclear what about the intermediate age range (2–4 months) makes data loss more important, bearing this in mind could be of some use when designing future studies.

**Figure 4:**
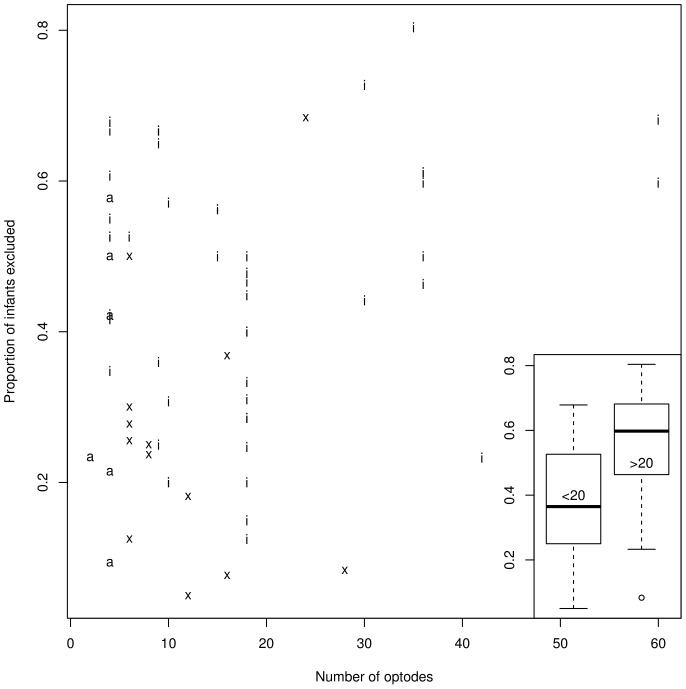
Proportion of infants excluded as a function of number of optodes. Number of optodes is totaled over sources and detectors. Each letter indicates an entry, coding for the system: ‘i’ stands for Hitachi, ‘a’ for Hamamatsu, and ‘x’ for all other systems. The boxplot on the right bottom panel represents the exact same data, collapsing into two categories: less than 20 optodes, and more than 20 optode.

Lloyd-Fox and colleagues have discussed the importance of instrumentation for reducing infant attrition and data loss, hypothesizing that technical advances leading to lighter and better-fitting headgear would result in lower attrition rates [3]. The current data, represented in Figure 4, suggests a modulation of this conclusion. It is certainly the case that having more than 20 optodes seems to be associated with higher attrition rates (median 54%; interquartile range 46%–68%). Thus, current expanses towards higher densities are being done at the expense of higher attrition rates. Furthermore, it should be noted that the attrition rate remains considerable even for studies using fewer than 19 optodes (median 36%; interquartile range 25%–52%). In other words, even with lighter headgears, more than a quarter of infants are typically excluded. Finally, in these data there was no indication of a reduction of attrition rate over the past 14 years.

Our database design included two more factors whose effect on attrition we had hoped to investigate. One of them related to study design, and involved the compromise of longer and more repetitive stimuli presentation to get better signal-to-noise ratios and higher attrition rates due precisely to this study design feature. We will breach those findings after introducing our results on the hemodynamic response in the next section. The other factor was the kind of optode tip used, indirectly coded through the system. Broadly speaking, there are at present two types of optode tips. One, exclusive to Hitachi, is pointed, and can be maneuvered around hair so that the tip touches the scalp. The other is flat, and may be affected to a greater extent by the presence of hair, although some believe it is more comfortable to wear. The latter type is not exclusive to any brand. By comparing systems in terms of attrition rate, one may begin to assess whether there is a difference of these two optode systems. However, this turned out to be impossible firstly because Hitachi does include flat tips in some caps. Secondly, the sheer imbalance in the prevalence of different systems make it difficult to draw any conclusions, as some effects (or lack thereof) could be due to the difference in the number of cases representing each system. In any case, an inspection of Figure 4 above does not reveal any clear patterns regarding whether certain systems (and their typical headgear) lead to lower attrition rates. This is certainly a topic worthy of quantitative investigation. It is possible that a different coding scheme may allow us to assess the impact of different headgear features, but this will not solve the ‘problem’ that most labs have opted for one system, and thus differences across systems will be difficult to assess.

### 3. Case study 2: Reliability of different hemoglobin measures

One of the unique features of fNIRS is that using multiple wavelengths allows one, in theory, to have independent estimates for both oxygenated hemoglobin (henceforth, oxy-Hb) and deoxygenated hemoglobin (henceforth, deoxy-Hb). A wealth of research has demonstrated that stimulation is followed by increases in oxy-Hb concentration and decreases in deoxy-Hb concentration [10]. There is an ongoing theoretical debate regarding whether current infant fNIRS research should focus on the increase in oxy-Hb, the decrease in deoxy-Hb, or an increase in total hemoglobin [oxy-Hb + deoxy-Hb] (see [4], for a recent discussion). We therefore assessed what is the empirical state of the art in infant fNIRS research.

Ideally, one would code for effect size in each of these measures. This was not possible given the markedly variable way in which results are reported. The only feasible coding we could implement was a ternary term for whether stimulation resulted in oxy-Hb increase, decrease, or no clear change; and the same for deoxy-Hb and total-Hb. Notice that we did not require this change to be prevalent in all infants and channels, or supported by statistics. It was simply based on statements by the authors, or visual inspection of figures showing average waveforms. Total-Hb is reported more rarely (35%); deoxy-Hb more frequently (67%), and oxy-Hb very commonly (85%). This need not reflect reliability of results, as it may be due to a bias that is reinforced within the field. Nonetheless, it does mean that results in total hemoglobin are probably not representative, and we will concentrate on oxy-Hb and deoxy-Hb in turn.

Oxy-Hb increased after stimulation in most of the entries (81 out of 95, or 85%); in the remaining 15%, half reported oxy-Hb decreasing and the other half no clear change. Since there is little variability in results, it is difficult to interrogate possible moderating factors. Nonetheless, DBIfNIRS does offer one lead, which could be investigated through more specific research. All of the oxy-Hb decreases were reported with high lower-end wavelengths (770–780 nm). Lower-end wavelengths are typically absorbed to a greater extent by deoxy-Hb. Thus, it is possible that the independent estimation of oxy-Hb and deoxy-Hb is less accurate at these wavelengths; a behavior deemed erratic for oxy-Hb may be recorded if it is confounded with deoxy-Hb. Indeed, some have argued for using low lower-end wavelengths, although the controversy remains because by increasing the separation between lower-end and higher-end wavelengths, they come to tap slightly different volumes [11]. There was no clear association with infant age or state, higher-end wavelength frequency, system, power, channel separation, or cortical area targeted.

Deoxy-Hb decreased after stimulation in about half of the entries (35 out of 74, or 47%), did not show a clear change in the other half (31/74, 42%), and increased rarely (8/74, 9%). Whether deoxy-Hb changes were positive, negative, or unclear seemed to be evenly distributed across infant age or state, higher-end wavelength frequencies, system, channel separation, or cortical area targeted. As with oxy-Hb, results going against expectation were more prevalent with high lower-end wavelengths, since 6 of the 8 deoxy-Hb increases were recorded using wavelengths 775 nm and higher. In addition, there may be a trend for deoxy-Hb results being more consistent with lower powers. Among the 32 entries that included details for power per source, 20 used powers below 1.2 mW, of which 85% yielded deoxy-Hb decreases and none increases. In the remaining 12 using powers 1.5 mW or higher, the percentage of results fitting the expected pattern was closer to 20%, with 36% of the entries actually bearing deoxy-Hb increases. Although a previous study comparing 0.6 and 1.2 mW had also concluded that the former resulted in higher sensitivity [7], only in 1 out of 8 measurements did they find a *reversed* response (see Fig. 4, p. 457). Future work could more specifically assess why higher power may actually lead to lower signal to noise, but we suggest that a reason why this affects deoxy-Hb (and not oxy-Hb) lies in the typical size of effects. Deoxy-Hb concentrations are known to be generally smaller in magnitude than oxy-Hb concentrations, and thus may be more likely to be buried in noise. Some unpublished observations of ours suggested that higher power may increase the levels of instrumentation noise in infants with little or no hair, as the power of the light could become too great and saturate the detectors. This is certainly a question to be explored with *ad hoc* designs.

### 4. Case study 3: Optimal designs for signal-to-noise ratios

One topic we hoped to explore related to the experiment duration, which in blocked designs results from a combination of stimulation duration, interstimulus interval duration, and the number of blocks infants are required to complete. Some members of the community believe that longer stimulation and interstimulus duration lead to better results. This is probably reasonable if the hemodynamic response varies across individuals more in its onset/offset characteristics, and/or typically has a pronounced initial dip; and it is probably best to have long blocks when using a general linear model, as they allow more accurate estimation of baseline levels and betas. Regardless of the analysis method, more blocks will always mean more measurements and (provided that infants stay still) higher signal to noise ratios. However, long and repetitive blocks are likely to result in restless infants, and they may result in weaker average activation levels due to habituation. Therefore, when designing a study, it would be important to have a rough approximation of what is a good compromise between these two goals. We calculated a number of measures that would be relevant to this question, including a proxy for study duration or amount of data as the duration of block plus baseline multiplied by the minimum number of blocks. In terms of data quality, we did the sum of oxy-Hb and deoxy-Hb patterns; a 2 would indicate a study with a typical hemodynamic response, a −2 one with an inverted response. Our hope was that we would find a minimum duration of stimulation, a minimum interstimulus interval, and a minimum number of blocks that virtually ensured a normal hemodynamic response, with as low an attrition rate as possible.

Unfortunately, we saw no clear patterns in the current DBIfNIRS one way or another. This may have to do with the difficulty of defining stimulation and baseline in cases where ‘blocks’ consist of a train of stimulation; or it may be due to the crude measures of signal-to-noise ratio used (a ternary division for each of oxy-Hb and deoxy-Hb). In addition, some data would need to be reported more consistently, such as how many blocks infants had to complete in order to be included (reported in about 60% of the entries). This is one area in which the field as a whole needs to contribute, by becoming more consistent in terms of the variables reported. In section 5, we lay out guidelines as to how this could be done.

### 4.5. Analyses and results

A sample of studies were coded by the first author in terms of their analyses methods and results. This involved 10 entries corresponding to the oldest and 10 for the most recent publications, in addition to a few others selected based on the first author's judgment of variability in reporting. In brief summary, this sample demonstrated that recent studies tend to pre-process data more, usually filtering, detrending, and removing artifacts. This pre-processing is documented with variable degrees of precision across different studies, and the precise criteria differed in nearly every paper read. Finally, methods for establishing activation were also markedly variable. These are clearly aspects that could be improved upon by more precise reporting in the future.

## Conclusions

In this paper, we presented the database DBIfNIRS, and assessed its utility by presenting the factors relating to three variables: infant attrition rate, the reliability of oxygenated and deoxygenated responses, and signal-to-noise ratios. Analyses of the first two variables illustrated the strengths of DBIfNIRS, while the signal-to-noise ratio analysis illustrated the limitations of the database. We found that the attrition rates of 2- to 4-month-olds are usually higher than newborns or 5- to 8-month-olds, suggesting that at certain points of development infants are ideally still and alert to facilitate fNIRS testing. Analyses of attrition rate in relation to the number of optodes suggested that infant attrition rate may also depend on the lightness of headgear. In studies where headsets had more than 20 optodes, the attrition rates were inordinately high, such that less than half of the infants could be included in the final analyses. It should be noted that the attrition rates of studies with fewer than 19 optodes were also considerable: In most studies, more than a quarter of infants tested were excluded. In our case study of reliability of different hemoglobin measures, we found that most of the erratic behaviors of oxy-Hb and deoxy-Hb were reported with high lower-end wavelengths of about 770–780 nm, and possibly with powers above 1.5 mW per physical source. As for signal-to-noise ratios, no clear patterns emerged from the inspection of available data, partially due to inconsistencies in data reporting and the difficulty of deciding on how to measure 'signal' and 'noise' in published research.

The results of our case studies underlined some relevant variables that could affect data quality. Therefore, analyses of published studies using DBIfNIRS could provide some insights to help fNIRS researchers make methodological choices when designing future studies. Furthermore, an online resource could keep the community informed not only in terms of methodology, but potentially also in terms of contents or research themes. Inputting one's study in the DBIfNIRS, and seeing others' studies, would provide timely information on who's working in one's field, and facilitate cooperation. Another advantage of the DBIfNIRS is that if producers of infant fNIRS literature enter their own data, the research community might be able to access information which is not reported in their papers. Finally, by virtue of being a community resource, the database does not rely on one or a few individuals micro-managing it. Instead, DBIfNIRS can grow with the help of many, while remaining trustworthy thanks to the supervision of a few expert volunteers.

Whilst there are numerous positive features in DBIfNIRS, there are also some limitations. To begin with, it is almost impossible to avoid errors when inputting manually such large amounts of information. In the immediate future, this problem is addressed by ensuring a minimal supervision, to be done by experts who volunteer to manage the database for two years. In the longer term, however, it would be ideal that harvesting these data were done automatically from papers producing standardized reports, such as those outlined below. A second problem is overtesting, which affects not only DBIfNIRS, but any other meta-analytic resource. In this paper, we have concluded that e.g. optode number affected attrition rate, a difference that was highly significant. However, if everyone does *t*-test after *t*-test on the same database, significance values become more difficult to interpret. Thus, it should be clear from the start that this database is a first approach, a ‘discovery sample’ of sorts, and should be complemented with more principled analyses and *ad hoc* hypothesis testing. A final potential problem is that the focus of the field changes; as new variables become important, other variables in the database could fade into the background. Our system of supervision allows DBIfNIRS to stay abreast of the field, as major structural changes can be implemented if needed.

A major contribution of DBIfNIRS is that it outlines several aspects of our data that cannot be a part of the database because they need to be more consistently reported. We propose a set of *strongly recommended and additional reporting guidelines*, shown on Table 2.

**Table 2:** Guidelines for infant fNIRS reporting.

	Strongly recommmended	Additional
Participants	Broad infant population type, state (involving sleep or awake), number included and excluded, criteria for exclusion, mean and range of chronological age, sex composition, language background in case of language study	Hair composition, skin pigmentation composition, (handedness composition of parents, for studies on lateralization), mean and range of gestational age at birth, whether sleep state is quiet sleep or active sleep in the case of sleeping infants
Instrumentation	Type of NIRS (e.g. CW system, time-resolved spectroscopy), Brand and model, power (mW per physical source), number of sources, number of detectors, number of channels defined, interoptode separation(s), sampling rate	Pad geometry, pad localization relative to an anchor in 10–20 space, mean and range of any head measurements that have been taken
Stimuli and procedure	General type of stimulation, general characteristics of baseline, mean and range of stimulation duration, mean and range of preceding and following rest duration, mean and range of number of 'good' stimulation blocks an infant*channel must have to be included	Total duration of the study, hyperlink to where (a sample of) the stimuli are stored
Pre-processing	Specific details on data processing for pre-processing, artifact detection, and removal, as detailed below. In all cases, specify to which signal they were applied; and whether they were applied by channel or channel group (e.g., optode- or pad-based). If relevant, analysis package used (e.g. HOMeR, POTATo)	Hyperlink to the scripts used throughout the pre-processing pipeline, if possible POTATo ‘recipe’ [12]
	1. Pre-processing and detrending: if filtering used, frequency and type of filter for low and band-pass; if baseline level fit, length of the preceding and following baseline, and type of fitting; in GLM, periods of sines/cosines declared for detrending	
	2. Artifact detection: for methods based on rapid changes, whether identification was automatic or manual, window size in seconds and criterion change; for PCA-based methods, method for selecting the principal component	
	3. Artifact removal: whether the whole block is excluded, only the artifacted stretch is removed, or whether the artifacted stretch is replaced (if so, what specific type of interpolation)	
Activation analyses and results	Report both oxyHb and deoxyHb; if using an ROI, explain how it was selected; if restricting the analyses to a temporal window, explain how it was selected; state which method was used to derive the dependent measure: GLM, average, peak (standard or absolute?), area under the curve; note if any further processing (e.g., z-scoring) was done to the data. Include a figure with the average time course and SE bars of oxyHb and deoxyHb changes for each channel	Hyperlink to the scripts used throughout the analysis pipeline, method of spatial estimation of brain region (e.g. photogrametry, 3D digitizer), hyperlink to where the original data are stored in a common format (e.g., if possible.snirf, [13])
Additional standardized signal-to-noise estimation	Using denoised data (but without z-scoring or converting it using absolute first), calculate, for each individual infant, oxy-Hb and deoxy-Hb peak amplitude within a time window starting at the onset of stimulation and ending at the shortest interstimulus interval used; within all channels in a ROI which plausibly includes a brain region that responds to the kind of stimulation used. Extract also the time of this peak response. Calculate the standard deviation across infants for this peak response for each individual channel. Then determine which of these channels has the greatest signal strength defined as the average peak amplitude divided by the standard deviation across infants for oxyHb and deoxyHb – this will be the *selected channel*. If the greatest signal strength according to oxyHb and deoxyHb is found in different channels, select both. For the selected channel(s) report: precise interoptode separation, localization relative to an anchor in 10–20 space, mean peak oxyHb, SD peak oxyHb, mean and SD latency of this peak oxyHb, mean peak deoxyHb, SD peak deoxyHb, mean and SD latency of this peak deoxyHb	

Items in 'strongly recommended' are necessary to achieve basic standardization and thus we should strive to report them; those in 'additional' would also be included for completeness.

Following a set of guidelines such as these would increase comparability across studies, and facilitate the detection of more or less favorable settings. While this is desirable, standardization is most likely to be achieved if it is based on items that are widely agreed upon within the community. To promote this crucial discussion, we have created a poll where participants can express their (dis)agreement with each of these items, and suggest additions or modifications. This poll, and its results, are available on the blog associated to DBIfNIRS. Although DBIfNIRS is already available, we foresee implementing some developments in the near future subject to opinions and responses of the user pool. Thanks to new technologies, DBIfNIRS can be useful in its present form at the same time as it is being improved upon. Moreover, it can be used as a forum within which key questions are discussed at the community level. Until consensus is reached, we look forward to other research teams utilizing DBIfNIRS to draw new methodological insights within infant fNIRS research.

## Supporting Information

Figure S1 **PRISMA flowchart.**
(PDF)

Table S1 **PRISMA checklist.**
(PDF)
