# Supplementary material for: An Online Database of Infant Functional Near InfraRed Spectroscopy Studies: A Community-Augmented Systematic Review
Source: PLoS One. 2013 Mar 15;8(3):e58906. doi: 10.1371/journal.pone.0058906 (PMC3598807; doi:10.1371/journal.pone.0058906)
Supplement: Figure S1 — PRISMA flowchart. (PDF) [file pone.0058906.s001.pdf]

PRISMA 2009 Flow Diagram

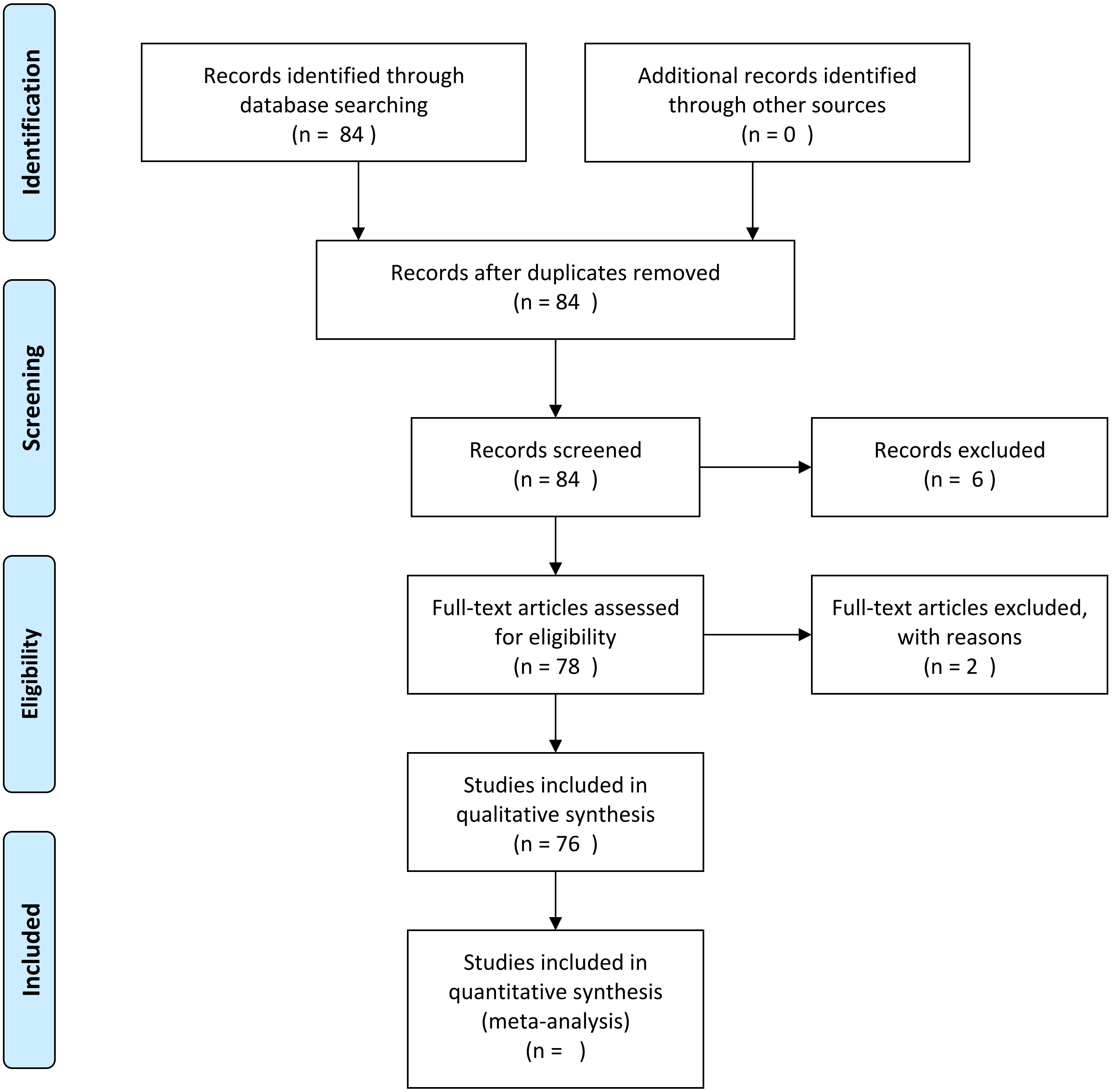

From: Moher D, Liberati A, Tetzlaff J, Altman DG, The PRISMA Group (2009). *P*referred *R*eporting *I*tems for *S*ystematic Reviews and *M*eta-*A*nalyses: The PRISMA Statement. PLoS Med 6(6): e1000097. doi:10.1371/journal.pmed1000097

For more information, visit www.prisma-statement.org.
